# Anticancer, antithrombotic, antityrosinase, and anti‐α‐glucosidase activities of selected wild and commercial mushrooms from Pakistan

**DOI:** 10.1002/fsn3.781

**Published:** 2018-09-14

**Authors:** Sumaira Sharif, Asia Atta, Tayyaba Huma, Asad Ali Shah, Gulnaz Afzal, Saira Rashid, Muhammad Shahid, Ghulam Mustafa

**Affiliations:** ^1^ Department of Biochemistry and Biotechnology University of Gujrat Gujrat Pakistan; ^2^ Department of Biochemistry Bahauddin Zakariya University Multan Pakistan; ^3^ Department of Bioinformatics and Biotechnology Government College University Faisalabad Pakistan; ^4^ Department of Life Sciences The Islamia University of Bahawalpur Bahawalpur Pakistan; ^5^ Department of Biochemistry University of Agriculture Faisalabad Faisalabad Pakistan; ^6^ Department of Biochemistry Government College University Faisalabad Pakistan

**Keywords:** anticancer, antithrombotic, antityrosinase, anti‐α‐glucosidase, mushrooms

## Abstract

Mushrooms have been accepted as nutraceutical foods because of their high nutritional and functional values. They have also gained interest due to their medicinal properties, economic importance, and organoleptic merit. In this study, wild *Ganoderma lucidum* and four commercial mushrooms, that is, *Pleurotus ostreatus, Volvariella volvacea, Hericium erinaceus,* and *Lentinus edodes* from Pakistan were screened for their biological activities such as anticancer, antityrosinase, anti‐α‐glucosidase, and antithrombotic activities from their methanol, ethanol, and water extracts. Enzyme inhibition assay showed that selected mushrooms are potent inhibitors with %age inhibition ranging from 19.00% to 80.91%, and 32.85% to 83.38% for tyrosinase and α‐glucosidase, respectively. The best tyrosinase inhibition was shown by *P. ostreatus* whereas *L. edodes* was found best as α‐glucosidase inhibitor. These mushrooms were tested against cancer cell lines including HT‐29 colon and H‐1299 lungs carcinoma cell lines. *G. lucidum* showed 29% and 24% viability of cells against HT‐29 and H‐1299 cell lines, respectively. This antiproliferative effect was in dose‐dependent manner, and the maximum inhibition was observed at 200 μg/ml. Mushrooms extracts were also found effective against clot lysis. The percentage of clot lysis was in the range of 27%–29%. The research would provide knowledge to the people of Pakistan about the importance of locally available commercial mushrooms and wild mushrooms for health improvement and prevention against different kinds of diseases.

## 1 INTRODUCTION

One of the most apparent influences of recent times is that people have brought their understanding back to the basics and to the natural or organic sources. Although the improvements brought by technology has made life relaxed to the people, but many are still looking for improved herbal substitutes that are proved to be more effective in their utmost natural form (Sanodiya, Thakura, Baghela, Prasadb, & Bisen, 2009). This matter had set the ideology of functional and nutraceuticals as the food that exerts beneficial effects beyond nutrition thereby reducing various ailments (El Sohaimy, 2012).

According to the International Agency for Research on Cancer (IARC), 148,041 people were reported to have cancer in 2012 among which death cases were about 101,113 in Pakistan (Sarwar & Saqib, 2017). The global increase of cancer incidence has been estimated by GLOBOCAN, an international agency for cancer research and it reported 12.7 and 8.2 million deaths in 2008 and 2012, respectively, and 14.1 million new cases in 2012, 64% of which belonged to developed countries due to cancer causing behavioral life style especially smoking (Ferlay et al., 2010, 2015). Currently available anticancer drugs are not target specific and pose several side effects and some complications in clinical management which encourage the urgent need for novel, effective, and nontoxic therapeutic approaches.

According to the World Health Organization (WHO), cardiovascular diseases (CVDs) (acute myocardial infarction and peripheral arterial thrombosis) are the causes of approximately 30% of deaths worldwide (Palomo, Fuentes, Padro, & Badimon, 2012). At this time, available thrombolytic agents are tissue plasminogen activator (t‐PA), streptokinase (SK), and urokinase (UK) that might cause serious bleeding complications along with reinfarction and reoccolution. Recently, a number of bioactive compounds from natural sources have been explored and identified as inhibitors to various cancerous cells and are safe alternative to treat cardiovascular and diabetes (Patel & Goyal, 2012). The search for new anticancer agents resulted in the isolation and purification of number of bioactive compounds from various mushroom species that were shown to have antitumor activity (Borchers, Krishnamurthy, Keen, Meyers, & Gershwin, 2008).

This study was aimed at the exploration of the potential of selected mushroom against cancer and cardiovascular diseases.

## 2 MATERIALS AND METHODS

### 2.1 Sample collection and preparation

The wild locally grown *Ganoderma lucidum* (Fr.) P. Karst., was isolated from the stem of *Salmalia malabarica* plant collected from Jinah garden, Faisalabad. Commercial locally cultivated *Pleurotus ostreatus* (Jacq. Ex. Fr.) Kumm., *Volvariella volvacea* (Bull, ex. Fr.) Sing., were collected from Horticulture Department, University of Agriculture Faisalabad, and exotic commercially available mushrooms *Lentinus edodes* (Berk.) Sing. and *Hericium erinaceus* (Bull.) Pers. (imported from China) were collected from local market. All the selected mushrooms were collected in dry form. Taxonomic identification was made by Prof. Dr. M. Asif Ali from medicinal mushroom lab, Institute of Horticultural Sciences, University of Agriculture Faisalabad, Pakistan. The specimen of each species was grounded in a domestic blender and reduced to fine dried powder and stored at 4°C before the extractions.

### 2.2 Extraction of selected mushrooms

The selected mushrooms were extracted in methanol, ethanol, and water according to the method given by Gangadevi, Yogeswari, Kamalraj, Rani, and Muthumary (2008) with slight modification. Briefly, dried mushroom powder 20 g was extracted with 200 ml of methanol, ethanol (80%), and water using an orbital shaker (Gallenkamp, UK) for 8 hr at room temperature. The extracts were separated from solid residue by filtering through Whatman No. 1 filter paper. The extract was evaporated in rotary evaporator (EYELA, N‐N Series; Rikakikai Co. Ltd. Japan) to yield the residue and stored at 4°C for subsequent analysis.

### 2.3 Anticancer potential of selected mushrooms

The in vitro cell proliferation assay was conducted as described by Jeff et al. (2013). The number of living cells at the end of incubation period was determined by colorimetric assay based on the tetrazolium salt MTT. In this assay, the tested samples were compared with control (without sample). All the experiments were performed in triplicate, and cell proliferation under each condition was expressed as a percentage of the control, which was set at 100%. All in vitro results were expressed as the proliferation ratio of tumor cells calculated as follows:

$$\text{Growth inhibition ratio}\;\left(\%\right)=\frac{1-B}{A}\times 100$$

where *A* and *B* are the average numbers of viable tumor cells for the control and samples, respectively, (Jeff et al., 2013).

### 2.4 α‐Glucosidase inhibition activity

The α‐glucosidase inhibition activity was performed according to the slightly modified method of Kwon, Apostolidis, and Shetty (2008) and Dong, Li, Zhu, Liu, and Huang (2012). Total volume of the reaction mixture of 100 μl contained 70 μl 50 mm phosphate buffer saline, pH 6.8, 10 μl (0.5 mm) test compound, followed by the addition of 10 μl (0.057 units) enzyme. The contents were mixed, preincubated for 10 min at 37°C, and preread at 400 nm. The reaction was initiated by the addition of 10 μl of 0.5 mm substrate (p‐nitrophenyl glucopyranoside). Acarbose was used as a positive control. After 30 min of incubation at 37°C, absorbance was taken at 400 nm using microplate reader (BioTek‐USA). The percent inhibition was calculated by the following equation:

$$\text{Inhibition}\;\left(\%\right)=\frac{\text{(Abs. of control ‐ Abs. of test solution)}}{\text{Abs.of control}}\times 100$$

IC_50_ values (concentration at which there is 50% in enzyme catalyzed reaction) of compounds were calculated using EZ‐Fit Enzyme Kinetics Software (Perrella Scientific Inc. Amherst, USA).

### 2.5 Tyrosinase inhibition activity

The antityrosinase effect of mushrooms was determined by calculating the hydroxylation of L‐tyrosine to L‐DOPA. Inhibition assay was conducted in 96‐well microplates, a spectrophotometer reader was used to determine the absorbance at 490 nm. Kojic acid was used as a positive control. (Momtaz et al., 2008).

### 2.6 Thrombolytic activities of selected mushrooms extracts and fractions

Clot lysis activity was checked using different mushrooms extracts and fractions (Prasad et al., 2006). The streptokinase was used as a positive control for in vitro clot lysis. In commercially available lyophilized streptokinase (SK) vial, 5 ml phosphate buffered saline (PBS) was added and assorted properly. This suspension was used as a stock from which proper dilutions were made to examine the anticlot activity.

#### 2.6.1 Sample preparation

Each extract (10 mg) was suspended in 1 ml dimethylsulfoxide (1%), and the suspension was shaken vigorously on a vortex.

Blood samples of different healthy volunteers were collected from different hospitals and laboratories of Faisalabad, Pakistan. Venous blood was drawn from healthy human volunteers without a history of oral contraceptive or anticoagulant therapy and irrespective of gender. Blood (500 μl) was transferred to the previously weighed microcentrifuge tubes.

#### 2.6.2 Preparation of clot

Preweighed microcentrifuge tubes which contain blood were incubated at 37°C for 45 min. Blood clot was formed at the bottom of each centrifuge tube. The serum was removed without disrupting the clot. Clots were weighed again to calculate weight of clot before lysis. Weight of clots (Wc) was determined by taking the difference of weight of microcentrifuge tubes (Wm) containing clot and weight of empty microcentrifuge tubes (We).

W_c = _w_m–_w_e_

#### 2.6.3 Addition of mushrooms extracts and fractions

Each mushroom extract (100 μl) was added in tubes, where streptokinase and distilled water were applied as positive and negative controls, respectively. All the microcentrifuge‐tubes were again incubated at 37°C for 90 min. for clot lysis. Then, tubes were inverted and left overnight. Microcentrifuge tubes were taken out of the incubator and the fluid obtained after lysis along with the applied agents (extract, streptokinase, and distilled water) was removed carefully. Tubes were weighed to calculate the weight of clot after lysis. The weights of clots were determined by taking difference between weights of clot after lysis (W1) and weight of empty tubes (We).

W_c_ = W_l_–W_e_

Then, percentage of clot lysis activity of different mushrooms extracts was determined by the difference between weight of clots before (Wb) and after lysis (W1) dividing by weight of clot before lysis and multiplied by 100.

$$\text{Clot lysis}\;\left(\%\right)=\frac{W_{b}-W_{1}}{W_{b}}\times 100$$

#### 2.6.4 Effect of concentrations, incubation time, and amount of sample on clot lysis

Mushrooms extracts of different concentrations (1%, 0.3%, and 0.6%) and incubated at different time intervals (30, 60 and 90 min), and 30, 60, and 100 μl of each concentration (1%, 0.6%, and 0.3%) of mushroom extract were used to determine the effect on thrombolysis.

### 2.7 Statistical analysis

The results obtained were presented by means ± standard deviation.

## 3 RESULTS AND DISCUSSION

### 3.1 Anticancer potential of studied mushrooms

**Figure 1:**
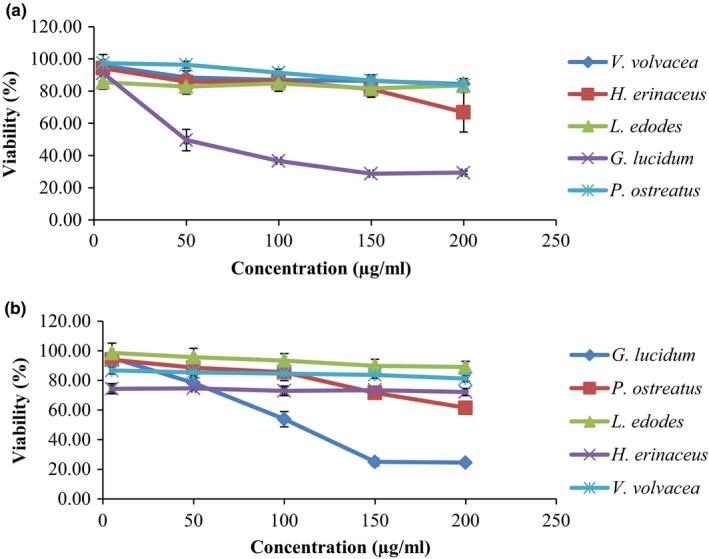
Viability (%) of water extracts of selected mushrooms at different concentrations against (a) colon tumor cells (HT‐29), (b) lung tumor cells (HT‐1299)

Cancer diseases are one of the main causes of death worldwide (Liu, Wang, Zhao, & Wang, 2013). The discovery of new molecules from natural origin is a global trend currently for the less toxicity of natural products (Wang et al., 2012). A number of bioactive compounds from natural resources had been investigated, identified, and isolated as inhibitor to various cancer cell lines (Ma, Chen, Dong, & Lu, 2013). In this study, the anticancer activity of water extracts of selected mushrooms was subjected to in vitro cytotoxicity assay in certain cancer cell lines including HT‐29 colon and H‐1299 lungs carcinoma cell lines. It was found that higher the concentration, the lower was the cell viability percentage (Thetsrimuang, Khammuang, Chiablaem, Srisomsap, & Sarnthima, 2011). The antiproliferative effect was in dose‐dependent manner, and the maximum inhibition was observed at the concentration of 200 μg/ml. The inhibitory activities of the water extracts on these cell lines are shown in Figure 1a,b.

All the mushrooms exhibited inhibition against HT‐29 cell lines. *G. lucidum* showed 29% viability of cells at 200 μg/ml followed by *H. erinaceous* 66%, *L. edodes* 68%, *V. volvacea* 83%, and *P. ostreatus* 84%. Whereas in case of H‐1299 cell lines, again the *G. lucidum* showed 24% viability of cells followed by *P. ostreatus* 61% and *H. erinaceus* 72%.

Jeff et al. (2013) found the β‐d‐glucan and monogalactoglucan isolated and purified from basidiocarps of *L. edodes* showed antitumor activity against S‐180, HCT‐116, and H‐29 cell lines with a dose‐dependent manner. In another in vivo study, the polysaccharide fraction extracted from the *Ganoderma* was shown to retard the growing sarcoma cells in mice (Hua et al., 2007). *G. lucidum* dried powder is recommended as a cancer chemotherapy agent in traditional Chinese medicine (TCM) and currently being utilized worldwide as dietary supplement (Stanley, Harvey, Slivova, Jiang, & Sliva, 2005).

It is concluded that the anticancer potential of *G. lucidum* might be due to high percentage of fiber contents that is 54% in our study. As dietary fibers are the carbohydrates in the diet that are not hydrolyzed by enzymes in either the stomach or small intestine, therefore they have importance for the management of different ailments. The anticancer potential of wild *G. lucidum* might be due to this attribute in addition to its antioxidant potential.

### 3.2 α‐glucosidase and tyrosinase inhibition activities of selected mushrooms

In the synthesis of melanin pigments, tyrosinases are responsible for coloring hairs, skin, and eyes and also for the treatments of some dermatological hyper pigmentation illness connected with overproductions of melanin (Chen, Ying, Li, & Yua, 2012). Skin hyper pigmentation can be depended on either an increased number of melanocytes or activity of melanogenic enzymes. Tyrosinase is a copper‐containing enzyme that catalyzes the oxidation of tyrosine into dopa and subsequently into dopaquinon. Natural inhibitors to tyrosinase such as mushrooms may consequently be functional and important in cosmetic business for skin whitening (Yoon, Alam, Lee, Lee, & Lee, 2011). The results from the above analysis showed that water extract of *P. ostreatus* is the best tyrosinase inhibitor (80.9%) among the selected commercial mushrooms followed by *L. edodes* (42.82%) whereas the *H. erinaceus* and *V. volvacea* exhibited less activities. Similarly, the IC_50_ values were lower for *P. ostreatus,* 34.78 μM as shown in Table 1.

**Table 1:** α‐glucosidase and tyrosinase inhibition activities of selected mushrooms water extracts (%DW)

Mushrooms	Antityrosinase activity		α‐glucosidase activity	
	Activity (%)	IC_50_ (μM)	Activity (%)	IC_50_ (μM)
*Lentinus* *edodes*	42.82 ± 0.69^c^	52.49 ± 0.95^b^	83.38 ± 0.91^d^	39.96 ± 0.74^a^
*Hericium erinaceus*	19.00 ± 0.17^a^	115.43 ± 1.02^d^	32.85 ± 1.04^a^	96.81 ± 0.83^d^
*Pleurotus ostreatus*	80.91 ± 0.82^d^	34.78 ± 0.92^a^	71.29 ± 1.17^c^	46.04 ± 0.81^b^
*Volvariella volvaceae*	25.4 ± 0.85^a^	89.61 ± 0.06^d^	36.74 ± 1.07^a^	82.4 ± 1.03^c^
*Ganoderma lucidum*	78.51 ± 0.26^d^	39.43 ± 0.89^a^	87.27 ± 0.87^d^	36.47 ± 0.82^a^
Standard	95.52 ± 0.46^d^	49.90 ± 0.12^b^	90.23 ± 0.14^d^	30.25 ± 0.46^a^

*Notes*

Standards used in this study along with their concentration: Acarbose (0.5 mm) α‐Glucosidase.

^a,b,c,d^Means ± *SD* followed by different superscripts in each row are significantly different at confidence level *p *≤* *0.05 using Tukey's multiple range test. Each value is a mean of three replicates.

Our results are in agreement with the study of Yoon et al. (2011), tyrosinase inhibitory activities of *L. edodes* acetonic, methanolic, and hot water extracts at concentration of 0.125–1 mg/ml were in the rage of 11.94% to 54.22%, 15.12% to 54.61%, and 3.09% to 47.32%, respectively. The inhibition of tyrosinase activity might be due to hydroxyl group of phenolic compounds of the mushrooms extracts that could form a hydrogen bond at the active site of enzyme, leading to a lower enzymatic activity (Baek et al., 2008).

α‐glucosidase delays the breakdown of carbohydrates in small intestine and diminishes the postprandial blood glucose excursion. It is effective and helps people with type 2‐diabetes, when blood sugar is elevated after eating complex carbohydrate. The results showed that α‐glucosidase inhibition activity was observed highest in *L. edodes* 83.38% and *P. ostreatus* 71.29% whereas moderate activity was observed in *H. erinaceus* and *V. volvacea*. IC_50_ values were also lower for *L. edodes* and *P. ostreatus* 39.96 and 46.4 μM, respectively, and can be compared with the positive control which showed the IC_50_ value 30.25%, whereas the IC_50_ values for *H. erinaceus* and *V. volvacea* were observed 96.81 and 82.4 μM, respectively.

Our results are consistent with Su, Lai, and Ng (2013); *n*‐hexane extract of *Grifola frondosa* showed a strong α‐glucosidase inhibitory activity. They also observed that α‐glucosidase inhibiting activity varied with the levels of oleic acid and linoleic acid present in the extracts. As a result of these properties, mushrooms could be used as natural food source for the management of blood glucose level in diabetic patients.

*G. lucidum* is best tyrosinase inhibitor and showed inhibition 78.51% with lower IC_50_ values 39.43 μM. The results also depict that α‐glucosidase inhibition activity was observed very high in *G. lucidum* 87.27% and as a result had lower IC_50_ values 36.47 μM. *G. lucidum* showed exceptionally high tyrosinase inhibition; this has led to its inclusion in many commercial skin‐whitening products and medical implication especially in relation to Parkinson disease (Chien, Tsai, Chen, Chang, & Tseng, 2008).

It is concluded from the above experiment that wild mushroom showed best tyrosinase and α‐glucosidase inhibition activities. Among the selected commercial mushrooms, locally cultivated *P. ostreatus* was the best tyrosinase and α‐glucosidase inhibitor as compared to the exotic commercial mushrooms.

### 3.3 Thrombolytic activity of mushrooms extracts and fractions

According to the World Health Organization (WHO), cardiovascular diseases (CVDs) (acute myocardial infarction, cerebrovascular disease, and peripheral arterial thrombosis) are the causes of approximately 30% of deaths worldwide (Palomo et al., 2012). In normal, body process coagulation and fibrinolysis processes are controlled properly. The dysfunction of fibrinolysis process or myocardial or cerebral infarction is a serious consequence of the thrombus formed in blood, and thus, blockage of blood vessels due to blood clot (fibrin clot) results in vascular disorders such as deep‐vein thrombosis, stroke, myocardial infarction, and pulmonary embolism (Choi et al., 2014). Thrombolytic agents are used to dissolve the already formed clots in the blood vessels (Ansari, Siddiqui, & Singh, 2012). Currently available thrombolytic agents are streptokinase (SK), tissue plasminogen activator (t‐PA), and urokinase (UK). They might cause serious bleeding complications along with reinfection and reoccolution and therefore secure and effective thrombolytic agents that can lyse a blood clot are desirable (Prasad et al., 2006).

The use of natural extracts in folk medicine suggests an economic and safe alternative to treat cardiovascular disease and infectious diseases. The clot lysis percentage for methanolic and ethanolic extracts was in the range from 9.4% to 27.4%. Thrombolytic activity of mushrooms extracts was determined to check the efficacy of natural extracts as thrombolytic agent. Results obtained are summarized in Tables 2.

**Table 2:** Thrombolytic activity of selected mushrooms ethanolic and methanolic extracts

Mushrooms	Solvents	% lysis
*Lentinus edodes*	Methanol	25.0
	Ethanol	27.4
*Pleurotus ostreatus*	Methanol	19.4
	Ethanol	16.1
*Hericium erinaceus*	Methanol	21.3
	Ethanol	27.9
*Volvariella volvacea*	Methanol	20.0
	Ethanol	16.2
*Ganoderma lucidum*	Methanol	12.8
	Ethanol	9.4
+ve control	Streptokinase	66.7
−ve control	Water	0.0

### 3.4 Effect of concentration, volume and time of incubation of selected mushrooms on thrombolysis

The percentage values of clot lysis of mushrooms extracts were directly proportional to concentrations, time of incubation, and amount of extract. The percentage values of clot lysis for *L. edodes* (ethanol) at concentrations of 0.3%, 0.6%, and 1% were 13.8%, 20.9%, and 27.4% respectively, and for *H. erinaceus* (ethanol) at 0.3%, 0.6%, and 1% were 16.7%, 18.4%, and 27.1%, respectively. The percentage values of clot lysis at different concentrations were ranged from 13% to 27% showing that percent lysis is directly proportional to concentration and the maximum activity was shown at 1% concentration. The results are presented in Table 3.

**Table 3:** Thrombolytic activity (% lysis) of ethanol extracts of selected mushrooms at three different levels of concentrations (0.3%, 0.6%, 1%), volume (30, 60, 100 μl) and incubation time (30, 60, 90 min)

Mushrooms (Ethanol)	Concentration (%)	% lysis
*Lentinus edodes*	0.3	13.8
	0.6	20.9
	1	27.4
*Hericium erinaceus*	0.3	16.7
	0.6	18.4
	1	27.1
*L. edodes*	Amount of extract (μl)	% lysis
	30	14.3
	60	16.5
	100	18.2
*Ganoderma lucidum*	30	7.4
	60	12.6
	100	17.1
*H. erinaceus*	30	11.1
	60	13.0
	100	16.9
*Volvariella volvacea*	Incubation time (min)	Absorbance (nm)
	30	0.596
	60	1.276
	90	1.568
*H. erinaceus*	30	1.326
	60	1.874
	90	1.953

*Lentinus edodes* (ethanol) at different amounts of 30, 60, and 100 μl were 14.3%, 16.5%, and 18.2%, respectively. For *G. lucidum* (ethanol) at 30, 60, and 100 μl, the values were 7.4%, 12.6%, and 17.1%, respectively, the percentage of clot lysis at different amounts were ranged from 7% to 18%. Ethanolic extracts of *H. erinaceus* showed the percentage values of clot lysis 11.11%, 13.0%, and 16.9% at 30, 60, and 100 μl, respectively. Increase in percentage clot lysis was observed by increasing amount of extract.

The absorbance for *H. erinaceus* (ethanol) recorded at different times of incubation at 30, 60, and 90 min were 1.326, 1.874, and 1.953 nm, respectively. The absorbance of clot lysis at each time of incubation differed from each other. In case of *V. volvacea* (ethanol), the absorbance values of clot lysis were 0.596, 1.276, and 1.568 nm, respectively, at 30, 60, and 90 min of incubation (Table 3). There was an increase in absorbance with increasing incubation time. The average percentage lysis values of natural extracts were close to synthetic compounds.

Up to our knowledge, no study has been conducted on thrombolytic potential of these selected mushrooms. This study showed that *L. edodes* and *H. erinaceus* extracts have significant thrombolytic activity as compared to the local cultivated mushrooms *P. ostreatus*. Wild *G. lucidum* also showed moderate thrombolytic potential.

## CONFLICT OF INTEREST

The authors declare that they do not have any conflict of interest.

## ETHICAL STATEMENT

This study does not involve any human or animal testing.
